# Eine retrospektive Identifikation von Schwerverletzten mittels ICD-10-Diagnosen: Teil des Projekts „Lebensqualität und Arbeitsfähigkeit nach schwerem Trauma“ (LeAf Trauma)

**DOI:** 10.1007/s00113-024-01446-w

**Published:** 2024-06-05

**Authors:** Anne Neubert, Sebastian Hempe, Veronika Gontscharuk, Carina Jaekel, Joachim Windolf, Erwin Kollig, Catharina Gäth, LeAf Trauma Study Group, Dan Bieler

**Affiliations:** 1 Klinik für Orthopädie und Unfallchirurgie, Medizinische Fakultät und Universitätsklinikum Düsseldorf, Heinrich-Heine-Universität Düsseldorf, Moorenstr. 5, 40225 Düsseldorf, Deutschland; 2 Klinik für Orthopädie und Unfallchirurgie, Wiederherstellungs- und Handchirurgie, Bundeswehrzentralkrankenhaus Koblenz, Koblenz, Deutschland; 3 Institut für Versorgungsforschung und Gesundheitsökonomie, Medizinische Fakultät und Universitätsklinikum Heine-Heine-Universität Düsseldorf, Düsseldorf, Deutschland; 4 Akademie für Unfallchirurgie (AUC), Deutsche Gesellschaft für Unfallchirurgie, Berlin, Deutschland

**Keywords:** Polytrauma, Krankenkassendaten, Injury Severity Score, Machine Learning, Retrospektive Analysen, Polytrauma, Health insurance data, Injury Severity Score, Machine Learning, Retrospective analyses

## Abstract

**Hintergrund:**

Durch eine stetige Verbesserung in der Behandlung überleben immer mehr Schwer- und Schwerstverletzte. Die Komplexität der Verletzungsmuster dieser Patient*innen bedingt, dass diese nur schwer in Routinedaten abbildbar sind.

**Ziel der Arbeit:**

Das Ziel der Auswertung war es, *International Statistical Classification of Diseases and Related Health Problems* (ICD-10)-Diagnosen, welche eine Assoziation mit einem Injury Severity Score (ISS) ≥ 16 aufweisen und somit zur Operationalisierung von Schwerverletzten in Routinedaten genutzt werden könnten, zu identifizieren.

**Material und Methoden:**

Es wurden die kodierten vierstelligen ICD-10-S-Diagnosen und der errechnete ISS von Traumapatienten des Bundeswehrzentralkrankenhauses Koblenz (BwZKrhs) und des Universitätsklinikums Düsseldorf (UKD) mittels statistischer Assoziationsmaße (Phi und Cramers V), linearer Regressionen sowie Methoden des *Machine Learning *(wie beispielsweise Random Forrest) analysiert.

**Ergebnisse:**

Es konnten S‑Diagnosen zu Gesichts‑, Kopf‑, Thorax- und Beckenverletzungen, die mit einem ISS ≥ 16 assoziiert waren, identifiziert werden. Manche S‑Diagnosen zeigten nur in einem der beiden Datensätze eine Assoziation mit einem ISS ≥ 16. Ebenso fanden sich assoziierte Gesichts‑, Kopf‑, Thorax- und Beckenverletzungen in der Subgruppenanalyse der 18- bis 55-Jährigen.

**Diskussion:**

Die aktuellen Auswertungen zeigen, dass es möglich ist, ICD-10-S-Diagnosen, welche eine signifikante Assoziation zu einem ISS ≥ 16 aufweisen, zu identifizieren. Gemäß dem Jahresbericht des TR-DGU® sind insbesondere in den Regionen Kopf und Thorax häufig Verletzungen mit einem *Abbreviated Injury Scale* Wert von ≥ 3 (AIS ≥ 3) zu finden.

**Graphic abstract:**

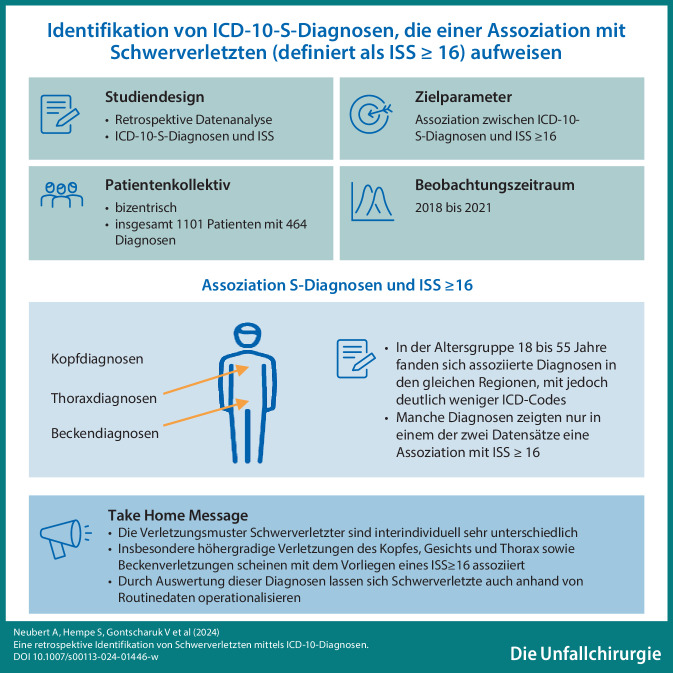

## Abstract (en)

**Background:**

Due to continuous improvements in treatment, more and more severely and seriously injured patients are surviving. The complexity of the injury patterns of these patients means that they are difficult to map in routine data.

**Aim of the work:**

The aim of the data exploration was to identify ICD 10 diagnoses that show an association with an injury severity score (ISS) ≥ 16 and could therefore be used to operationalize severely injured patients in routine data.

**Material and methods:**

The coded four-digit ICD 10 S diagnoses and the calculated ISS of trauma patients from the Armed Forces Central Hospital Koblenz (BwZKrhs) and the University Hospital Düsseldorf (UKD) were analyzed using statistical association measures (phi and Cramer’s V), linear regressions and machine learning methods (e.g., random forest).

**Results:**

The S diagnoses of facial, head, thoracic and pelvic injuries, associated with an ISS ≥ 16 were identified. Some S diagnoses showed an association with an ISS ≥ 16 in only 1 of the 2 datasets. Likewise, facial, head, thoracic and pelvic injuries were found in the subgroup of 18–55-year-old patients.

**Discussion:**

The current evaluations show that it is possible to identify ICD 10 S diagnoses that have a significant association with an ISS ≥ 16. According to the annual report of the trauma register of the German Society for Trauma Surgery (TR-DGU®), injuries with an abbreviated injury scale (AIS) ≥ 3 are particularly common in the head and thoracic regions.

## Einleitung

Die Versorgung von Schwerverletzten ist komplex und langwierig. Durch eine stetige Verbesserung in der Behandlung überleben immer mehr dieser Patient*innen. Die Komplexität des Verletzungsmusters dieser Patient*innen bedingt, dass diese nur schwer in Routinedaten abbildbar sind. Abhängig von dem zugrunde liegenden Verletzungsmuster und den durchgeführten Interventionen werden viele verschiedene Kodierungen der *International Statistical Classification of Diseases and Related Health Problems* (ICD-10) und des Operationen- und Prozedurenschlüssels (OPS) verwendet [1].

Im Rahmen der Datenübermittlung gemäß § 301 Fünftes Buch Sozialgesetzbuch (SGB V) sind die Übermittlung des *Injury Severity Score* (ISS) und damit die Übermittlung der Verletzungsschwere dieser Patient*innen nicht vorgesehen. Eine Erhebung dieser Daten erfolgt lediglich im Rahmen der Dateneingabe in das TraumaRegister® der Deutschen Gesellschaft für Unfallchirurgie (TR-DGU®). Dementsprechend sind eine retrospektive Identifizierung von schwer verletzten Patient*innen (ISS ≥ 16) sowie eine retrospektive Auswertung von entsprechenden Gesundheitsdaten aus den Routinedatensätzen dieser Patient*innen ohne gleichzeitigen Zugriff auf Daten aus dem TR-DGU® nicht möglich. Dies erschwert insbesondere Forschungsvorhaben zu Schwerverletzten deutlich.

Das Ziel der Datenexploration war, ICD-10-Diagnosen, welche eine Assoziation mit einem ISS ≥ 16 aufweisen und somit zur Operationalisierung von schwer verletzten Patient*innen in Routinedaten genutzt werden könnten, zu identifizieren. Somit könnte zukünftig eine verbesserte Untersuchung der Versorgung und auch des langfristigen Heilungsverlaufs von Schwerverletzten ermöglicht werden.

## Methodik

Eingeschlossen wurden Patient*innen des Bundeswehrzentralkrankenhauses Koblenz (BwZKrhs) der Jahre 2019–2021 und des Universitätsklinikums Düsseldorf (UKD) der Jahre 2018–2021, welche im TR-DGU® erfasst wurden. Für diese Patient*innen wurden alle kodierten vierstelligen ICD-10-S-Diagnosen aus den Datensätzen gemäß § 21 KHEntgG pseudonymisiert extrahiert. Die S‑Diagnosen wurden dann mit dem errechneten ISS der Patient*innen verknüpft. Die Analysen wurden mittels statistischer Assoziationsmaße (Phi und Cramers V), linearer Regressionen sowie Methoden des *Machine Learning *(wie beispielsweise Random Forrest) durchgeführt. Für das UKD wurde ein positives Ethikvotum der Heinrich-Heine-Universität Düsseldorf eingeholt (Studiennummer: 2022-2029_2). Für das BwZKrhs war im Einklang mit den in Rheinland-Pfalz geltenden Richtlinien kein Ethikvotum zur Analyse der retrospektiven Daten notwendig.

## Ergebnisse

Insgesamt wurden im BwZKrhs 598 Patient*innen mit insgesamt 259 S‑Diagnosen und im UKD 503 Patient*innen mit insgesamt 265 S‑Diagnosen erfasst. Bei Patient*innen mit einem ISS ≥ 16 (*n* = 613) fanden sich im BwZKrhs (*n* = 283) 212 S‑Diagnosen und im UKD (*n* = 330) 217 S‑Diagnosen. Bei ca. 75 % aller Patient*innen zeigten sich 3 oder mehr S‑Diagnosen im UKD und 2 oder mehr im BwZKrhs. Es zeigte sich eine hohe Vielfalt an S‑Diagnosen mit geringer Häufung in beiden Kliniken, sodass bei den Analysen ausschließlich die 10 % häufigsten S‑Diagnosen aus den Datensätzen im Fokus standen. Es konnten S‑Diagnosen zu Gesichts‑, Kopf‑, Thorax- und Beckenverletzungen, die mit einem ISS ≥ 16 assoziierten waren, identifiziert werden (Tab. 1).

**Tab. 1:** Assoziation zwischen ICD-10-S-Diagnosen und ISS > 16 (alle Altersgruppen/beiden Datensätze)

Körperregion	ICD-10-GM-Kodierung	Beschreibung
Gesichtsverletzungen	S02.1	Schädelbasisfraktur
	S02.3	Fraktur des Orbitabodens
Kopfverletzungen	S06.0	Gehirnerschütterung
	S06.1	Traumatisches Hirnödem
	S06.2	Diffuse Hirnverletzung
	S06.5	Traumatische subdurale Blutung
	S06.6	Traumatische subarachnoidale Blutung
Thoraxverletzungen	S22.0	Fraktur eines Brustwirbels
	S22.4	Rippenserienfraktur
	S27.0	Traumatischer Pneumothorax
	S27.3	Sonstige Verletzungen der Lungen
	S42.1	Fraktur der Skapula
Beckenverletzungen	S32.1	Fraktur des Os sacrum
	S32.5	Fraktur des Os pubis

Manche S‑Diagnosen zeigten nur in einem der beiden Datensätze eine Assoziation mit einem ISS ≥ 16. Dazu zählen im BwZKrhs die S‑Diagnosen für weitere Verletzungen des Gesichts und der Wirbelsäule. Im UKD waren es ICD-10-Kodierungen für weitere Verletzungen des Beckens.

Bei den Analysen zu der Altersgruppe 18 bis 55 Jahre (BwZKrhs: *n* = 275; UKD: *n* = 252) zeigten sich in beiden Datensätzen grundsätzlich weniger Diagnosen, die einen Zusammenhang mit einer schweren Verletzung (ISS ≥ 16) aufwiesen. Darunter fanden sich Gesichts‑, Kopf‑, Thorax- und Beckenverletzungen, wie in Tab. 2 dargestellt.

**Tab. 2:** Assoziation zwischen ICD-10-S-Diagnosen und ISS > 16 (Altersgruppe 18 bis 55 Jahre/beide Datensätze)

Körperregion	ICD-10-GM-Kodierung	Beschreibung
Gesichtsverletzungen	S02.1	Schädelbasisfraktur
Kopfverletzungen	S06.1	Traumatisches Hirnödem
Thoraxverletzungen	S22.4	Rippenserienfraktur
	S27.0	Traumatischer Pneumothorax
	S27.3	Sonstige Verletzungen der Lungen
	S42.1	Fraktur der Skapula
Beckenverletzungen	S32.1	Fraktur des Os sacrum
	S32.5	Fraktur des Os pubis

Weiterhin fanden sich in einem der beiden Datensätze Gesichts- (BwZKrhs: S02.0/S02.4/S02.6; UKD: S02.3), Kopf- (BwZKrhs: S06.2/S06.4/S06.5/S06.6), Becken-/Wirbelsäulen- (BwZKrhs: 32.0; UKD: S32.3/S32.4) sowie Abdomenverletzungen (BwZKrhs: S36.1; UKD: S36.0) assoziiert mit ISS ≥ 16.

## Diskussion

Zwar existieren diverse Umrechnungstabellen/-tools (ICD-AIS map, ICDPIC), die in einigen Fällen eine ungefähre Umrechnung von ICD-10 zur Abbreviated Injury Scale (AIS) und dem damit verbunden ISS zulassen, jedoch sind diese häufig nicht auf das deutsche Kodiersystem (ICD-10 GM) zugeschnitten und daher zu ungenau oder für die einfache Identifikation Schwerverletzter in großen Routinedatensätzen nicht praktikabel bzw. qualitativ unzureichend [2, 3].

Die aktuellen Auswertungen zeigen, dass es möglich ist, ICD-10-S-Diagnosen, welche eine signifikante Assoziation zu einem ISS ≥ 16 aufweisen, zu identifizieren. Gemäß dem Jahresbericht des TR-DGU® sind insbesondere in den Regionen Kopf und Thorax häufig Verletzungen mit einem AIS-Wert ≥ 3 zu finden [4]. Verletzungen in eben diesen Regionen zeigten auch in unserer Auswertung eine Assoziation zu einem ISS ≥ 16. Des Weiteren scheinen diese S‑Diagnosen auch aus klinischer Sicht wahrscheinlicher bei Schwerverletzten aufzutreten. Diese Diagnosen könnten es demnach ermöglichen, in Verbindung mit anderen Parametern aus Routinedatensätzen (z. B. Aufenthaltsdauer auf einer Intensivstation, Anzahl der Beatmungsstunden, oder kodierte OPS) neue Modelle zur Identifikation Schwerverletzter zu entwickeln.

## Schlussfolgerung

Es zeigt sich, dass, basierend auf den Datensätzen von zwei verschiedenen Krankenhäusern, plausible ICD-10-S-Diagnosen, die mit einer Verletzungsschwere ISS ≥ 16 assoziiert sind und somit charakteristisch für Schwerverletzte sein können, identifiziert werden können. Bedingt durch die Vielfalt an möglichen Diagnosen, die bei Schwerstverletzten auftreten können, zeigte sich auch in unseren Analysen eine große Vielfalt an S‑Diagnosen, von denen viele nur mit einer geringen Häufung aufgetreten sind. Dies schränkte die Analyse ein. In größeren Datensätzen aus weiteren Krankenhäusern lassen sich u. U. noch weitere S‑Diagnosen, die sich als charakteristisch für Schwerverletzte erweisen können, identifizieren. Limitierend sollte jedoch angeführt werden, dass bei den Analysen von ICD-10-Kodierungen immer bedacht werden muss, dass diese Kodierungen nicht vollumfänglich den Befund der Patient*innen widerspiegeln, da diese Kodierungen auch ökonomischen Interessen unterliegen. Dies kann bedingen, dass einige Diagnosen, die relevant für Einstufung eines Patienten als schwerstverletzt wären, nicht aufgeführt werden, da diese Kodierungen bei dem jeweiligen Patienten nicht abrechnungsrelevant sind.

## Fazit für die Praxis

- Die Verletzungsmuster Schwerverletzter sind interindividuell sehr unterschiedlich, weshalb häufig viele verschiedene ICD-10-S-Diagnosen kodiert werden.
- Insbesondere höhergradige Verletzungen des Kopfes, Gesichts und Thorax sowie Beckenverletzungen scheinen mit dem Vorliegen eines ISS ≥ 16 assoziiert zu sein.
- Die genannten S‑Diagnosen können es ermöglichen, Schwerverletzte anhand retrospektiver Auswertungen von Routinedatensätzen zu identifizieren und operationalisieren.

## Abkürzungen

AIS: Abbreviated Injury Scale
BwZKrhs: Bundeswehrzentralkrankenhaus Koblenz
ICD-10: International Statistical Classification of Diseases and Related Health Problems
ICD-10 GM: International Statistical Classification of Diseases and Related Health Problems – Deutsche Fassung
ISS: Injury Severity Score
OPS: Operationen- und Prozedurenschlüssel
SGB V: Fünftes Sozialgesetzbuch
TR-DGU®: TraumaRegister® der Deutschen Gesellschaft für Unfallchirurgie
UKD: Universitätsklinikum Düsseldorf
